# Causal effects of gut microbiota on the risk of chronic kidney disease: a Mendelian randomization study

**DOI:** 10.3389/fcimb.2023.1142140

**Published:** 2023-03-31

**Authors:** Mingli Luo, Jiahao Cai, Shulu Luo, Xiaosi Hong, Lingxin Xu, Honghong Lin, Xiong Chen, Wen Fu

**Affiliations:** ^1^ Department of Pediatric Urology, Guangzhou Women and Children’s Medical Center, Guangzhou Medical University, Guangdong Provincial Clinical Research Center for Child Health, Guangzhou, China; ^2^ Department of Urology, Sun Yat-sen Memorial Hospital, Sun Yat-sen University, Guangzhou, China; ^3^ Department of Neurology, Guangzhou Women and Children’s Medical Center, Guangzhou Medical University, Guangdong Provincial Clinical Research Center for Child Health, Guangzhou, China; ^4^ Department of Prosthodontics, Hospital of Stomatology, Guanghua School of Stomatology, Sun Yat-sen University, Guangzhou, China; ^5^ Department of Endocrinology, Sun Yat-Sen Memorial Hospital, Sun Yat-Sen University, Guangzhou, China; ^6^ Department of Radiation Oncology, Sun Yat-sen University Cancer Center, Guangzhou, China; ^7^ Department of Pediatric Orthopedics, Guangzhou Women and Children’s Medical Center, Guangzhou Medical University, Guangdong Provincial Clinical Research Center for Child Health, Guangzhou, China

**Keywords:** gut microbiota, chronic kidney disease, *Mendelian randomization*, CKD, nutrition

## Abstract

**Background:**

Previous studies have reported that gut microbiota is associated with an increased risk of chronic kidney disease (CKD) progression. However, whether gut microbiota has a causal effect on the development of CKD has not been revealed. Thus, we aimed to analyze the potential causal effect of gut microbiota on the risk of CKD using mendelian randomization (MR) study.

**Materials and Methods:**

Independent single nucleotide polymorphisms closely associated with 196 gut bacterial taxa (N = 18340) were identified as instrumental variables. Two-sample MR was performed to evaluate the causal effect of gut microbiota on CKD (N = 480698), including inverse-variance-weighted (IVW) method, weighted median method, MR-Egger, mode-based estimation and MR-PRESSO. The robustness of the estimation was tested by a series of sensitivity analyses including Cochran’s Q test, MR-Egger intercept analysis, leave-one-out analysis and funnel plot. Statistical powers were also calculated.

**Results:**

The genetically predicted higher abundance of order *Desulfovibrionales* was causally associated with an increased risk of CKD (odds ratio = 1.15, 95% confidence interval: 1.05-1.26; *p* = 0.0026). Besides, we also detected potential causalities between nine other taxa (*Eubacterium eligens group*, *Desulfovibrionaceae*, *Ruminococcaceae UCG-002*, *Deltaproteobacteria*, *Lachnospiraceae UCG-010*, *Senegalimassilia*, *Peptostreptococcaceae*, *Alcaligenaceae* and *Ruminococcus torques group*) and CKD (*p* < 0.05). No heterogeneity or pleiotropy was detected for significant estimates.

**Conclusion:**

We found that *Desulfovibrionales* and nine other taxa are associated with CKD, thus confirming that gut microbiota plays an important role in the pathogenesis of CKD. Our work also provides new potential indicators and targets for screening and prevention of CKD.

## 1 Introduction

Chronic kidney disease (CKD) is defined as renal structural abnormalities and dysfunction on account of multifarious causes for more than 3 months, which includes the following evidence: At least one renal injury marker is detected and (or) a decrease in glomerular filtration rate (GFR) to < 60mL/min·1.73m^2^ (Webster et al., 2017; Kalantar-Zadeh et al., 2021). Studies indicate that the incidence of CKD is increasing year by year and has become a huge burden of global public health (GBD Chronic Kidney Disease Collaboration, 2020; Webster et al., 2017). The global prevalence of CKD was estimated at 11-14%, and the latest data show that almost 10% of adults worldwide are suffering from CKD (GBD Chronic Kidney Disease Collaboration, 2020; Hill et al., 2016; Lv and Zhang, 2019; Kovesdy, 2022). To make matters worse, CKD also has a high mortality rate. As an essential etiology of chronic kidney failure and uremia, CKD is estimated to be responsible for 1.2 million death cases per year (Xie et al., 2018). Moreover, the number of deaths due to CKD is increasing rapidly at a rate second only to that caused by HIV infection (Webster et al., 2017). It is estimated that CKD would become the fifth leading cause of death worldwide in the coming 20 years (Foreman et al., 2018). Since the progression of CKD is irreversible, it is necessary to study the etiology of CKD in order to better prevent the occurrence of CKD.

Under normal circumstances, there are a large number of bacteria colonized harmoniously in the human gut, constituting a specific intestinal microecosystem (Milani et al., 2017). Gut microbiota has been proved to be involved in the regulation of human metabolism and immune activities, and play an important role in maintaining the homeostasis (Musso et al., 2010; Maynard et al., 2012; Frosali et al., 2015; Fujisaka et al., 2018). In addition, there is increasing evidence that gut microbiota is associated with the pathogenesis and progression of many diseases, such as metabolic diseases, autoimmune diseases and tumors (De Luca and Shoenfeld, 2019; Scheithauer et al., 2020; Matson et al., 2021). In recent years, with the introduction of the concept of gut-kidney axis, more and more attention has been paid to the role of gut microbiota in CKD (Meijers and Evenepoel, 2011). Previous studies have shown significant alterations in the abundance and structure of intestinal flora in patients with CKD, which is known as dysbiosis and leads to the disruption of the intestinal barrier (Wang et al., 2012; Vaziri et al., 2013). In turn, when the leaky gut is formed, the dysfunctional gut microbiota may induce systemic inflammation, oxidative stress and immune regulation disorder through the release of metabolites (such as lipopolysaccharide and enterogenous uremic toxins), and further aggravate the deterioration of renal function in patients with CKD (McIntyre et al., 2011; Shiba et al., 2014; Felizardo et al., 2016; Evenepoel et al., 2017; Kim and Song, 2020). These findings shed light on the association between gut microbiota and CKD progression, and suggest that gut microbiota may be a potential causal factor of CKD. However, to explore whether gut microbiota has a causal effect on CKD and to further identify which bacterial taxa increase the risk of CKD, a large number of rigorous biomedical and observational studies are still needed, which are costly, cumbersome and difficult to control (de la Torre Hernández and Edelman, 2017).

According to the law of independent assortment, genetic variants will be randomly assorted to gametes during meiosis. Mendelian randomization (MR) analysis is a research method that uses this law as a principle to simulate randomized controlled trial (RCT) using single nucleotide polymorphisms (SNPs) as genetic instrumental variables (IVs). Because of this, MR study is seen as an appropriate method for analyzing the causal effects of exposure on clinical outcomes, which circumvents the influence of confounding factors and is cost-effective (Emdin et al., 2017; Zheng et al., 2017). Since the microbiome does not alter an individual’s DNA sequence (Thomas, 2019), this property makes it feasible to analyze the causal relationship between gut microbiota and CKD with MR study.

Hence, our study attempted to reveal the potential causal effects of gut microbiota on the risk of CKD through MR analyses and to identify bacterial taxa that increase the risk of CKD. Ultimately, *Desulfovibrionales* and nine other taxa were found to be causally associated with CKD.

## 2 Methods

### 2.1 Study overview

**Figure 1:**
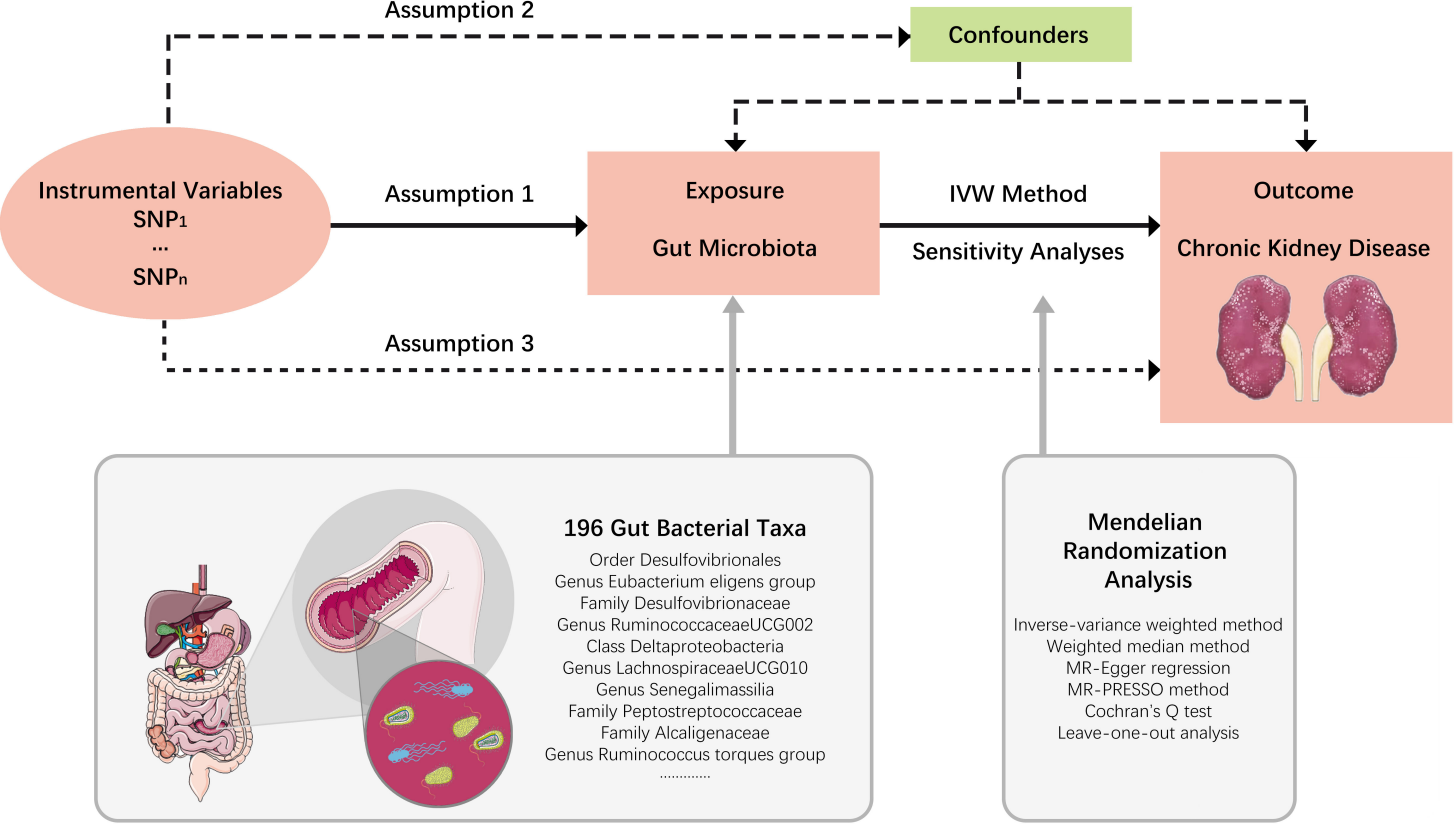
Study design. An overview of the study design. SNP, single nucleotide polymorphisms; IVW, inverse-variance weighted.

In this study, each bacterial taxon contained in the gut microbiota was categorized as a separate exposure. A two-sample MR analysis was performed to determine which bacterial taxa had a causal effect on CKD using summary statistics of genome-wide association studies (GWAS). An overview of the study design is shown in **Figure 1**, which includes three key assumptions that must be met in a MR study: 1) the genetic variant is associated with the exposure of interest; 2) the genetic variant is not associated with confounders; and 3) the genetic variant influences the outcome only through the exposure of interest (Emdin et al., 2017). The STROBE-MR guideline was used to guide the design of this study (Skrivankova et al., 2021), with the checklist available in the **Supplementary Material**.

### 2.2 Data source of CKD

For CKD, the SNP information was obtained from CKDGen Consortium, a global research collaboration that has conducted the largest genome-wide association meta-analysis of CKD to date. Taking sample size, sequencing depth, ethnicity and data update time into account, the genome-wide genetic dataset of kidney function published by Wuttke et al. in 2019 was selected for this study (Wuttke et al., 2019). The GWAS meta-analysis of CKD included in this dataset pooled more than 23 cohorts and included 41395 CKD cases and 439303 controls (n=480698 in total) (**Table S1**), all of whom were of European ancestry. All cases enrolled in this meta-analysis were diagnosed as CKD according to the criteria of estimated GFR < 60mL/min·1.73m^2^. Moreover, the GWAS summary statistics were adjusted by sex, age, body mass index and first 10 genetic principal components.

### 2.3 Selection of genetic instruments

The genetic IVs of each bacterial taxon were obtained from the largest meta-GWAS of human gut microbiota, which comprised 18340 individuals from 24 cohorts, 14363 of whom (>78%) were of European ancestry (**Table S2**) (Kurilshikov et al., 2021). The microbiome GWAS was adjusted by age, sex, technical covariates and genetic principal components. After removing 15 unknown bacterial taxa, the GWAS data we obtained finally covered a total of 196 taxa (sorted by taxonomy): 9 phyla, 16 classes, 20 orders, 32 families and 119 genera (**Table S3**) (Kurilshikov et al., 2021).

For each taxon, the SNPs were then filtered by the following steps: 1) a relaxed genome-wide significance threshold of *p* < 1×10^-5^ was adopted due to the limited number of SNPs available with genome-wide significance *p* < 5×10^-8^ (Sanna et al., 2019). 2) The Linkage disequilibrium (LD) test was performed using PLINK (v 1.9), and LD r^2^ < 0.1 within a window of 500 kb was adopted to ensure the independence of the selected IVs (Ni et al., 2021). For those SNPs missing from the outcome dataset, proxy-SNPs with LD r^2^ > 0.8 were used. 3) The F-statistic of each SNP was calculated and SNPs with *F* < 10 were eliminated to avoid weak instruments bias (Burgess and Thompson, 2011). 4) The SNPs that were incompatible, or palindromic with intermediate allele frequency, were removed in the process of harmonizing.

### 2.4 Mendelian randomization analysis and sensitivity analysis

In this study, inverse-variance-weighted (IVW) method was used as the main analysis to preliminarily evaluate the potential causal effects of each phenotype on CKD risk. Weighted median (WM) method, MR-Egger regression, Mendelian Randomization Pleiotropy Residual Sum and Outlier (MR-PRESSO) test and mode-based estimate (MBE) analysis were performed as robustness validation, whose results were complementary to those estimated by IVW method (Bowden et al., 2015; Bowden et al., 2016; Hartwig et al., 2017). For a certain phenotype, if the causal effects estimated by the five methods were inconsistent, a more stringent genome-wide significance threshold was used to reselect the IVs and recalculate the causal effects (Chen et al., 2021).

Potential heterogeneity was quantified and tested by calculating Cochran’s Q statistics, and horizontal pleiotropy was estimated by MR-Egger intercept test. Leave-one-out analysis was conducted to identify and remove any potential outliers that independently influence the observed causal relationship. For significant MR estimates, MR-PRESSO test was also used to detect any outliers and adjust for heterogeneity. If the heterogeneity was detected among the IVs, the outliers were removed and the MR analysis was performed all over again.

To eliminate the influence of confounding factors, we further searched the PhenoScanner (http://www.phenoscanner.medschl.cam.ac.uk) to check whether the selected SNPs of significant MR estimates in this study were associated with other CKD risk factors, which mainly concerning hypertension, diabetes, obesity, glomerulonephritis and carcinoma. After excluding the confounder-related SNPs, the causal effects were rechecked to determine if they remained significant. Since the robustness of MR analysis will be destroyed when sample overlap exists, an online tool (https://sb452.shinyapps.io/overlap/) was used to calculate bias and type I error rates for Mendelian randomization with samples overlap (Burgess et al., 2016).

### 2.5 Statistical analysis

Using Bonferroni correction, we established significance thresholds for the primary MR results at each feature level (phylum, class, order, family and genus). When there are n bacterial taxa included in a feature level, the Bonferroni-corrected significance threshold for that feature level should be 0.05/n. For example, for MR results of phylum, considering that there were 9 taxa contained in phylum level, a Bonferroni-corrected threshold of *p* value was set as 0.05/9 (5.56×10^-3^). Likewise, for MR results of class, order, family and genus, the Bonferroni-corrected thresholds of *p* value were 3.13×10^-3^, 2.5×10^-3^, 1.56×10^-3^ and 4.20×10^-4^, respectively. MR results with *p* values less than Bonferroni-corrected threshold could be considered significant. Meanwhile, we considered MR estimates with *p* < 0.05 as nominal significant. The *p* values of other test reports in this study were all two-tailed, and *p* < 0.05 could be regarded as a significant difference.

All analyses above were mainly completed by using the Two-Sample-MR package (version 0.5.5) of R software (version 4.0.2). Moreover, an online power calculation tool for MR (https://shiny.cnsgenomics.com/mRnd/) was used to calculate the statistical power of causal effect estimates (Burgess, 2014). A power threshold of 0.8 was considered appropriate so that 4/5 of the false null hypothesis would be rejected (Fox and Mathers, 1997).

## 3 Results

### 3.1 An overview of IVs in taxa

Through screening the genome-wide significance threshold (*p* < 1×10^-5^), LD test, harmonizing, MR-PRESSO test and verifying *F* statistics, each of 196 bacterial taxa got multiple SNPs (ranging from 3 to 22) as their proxies. SNPs detected as outliers by MR-PRESSO (global test: *p* < 0.05) were all removed. The *F* statistics of all retained SNPs were over 10, indicating sufficient correlation strength between IVs and corresponding bacterial taxon. See **Table S4** for the final list of retained SNPs and relevant statistical data.

### 3.2 Associations of genetically proxied gut bacterial taxa with CKD

**Figure 2:**
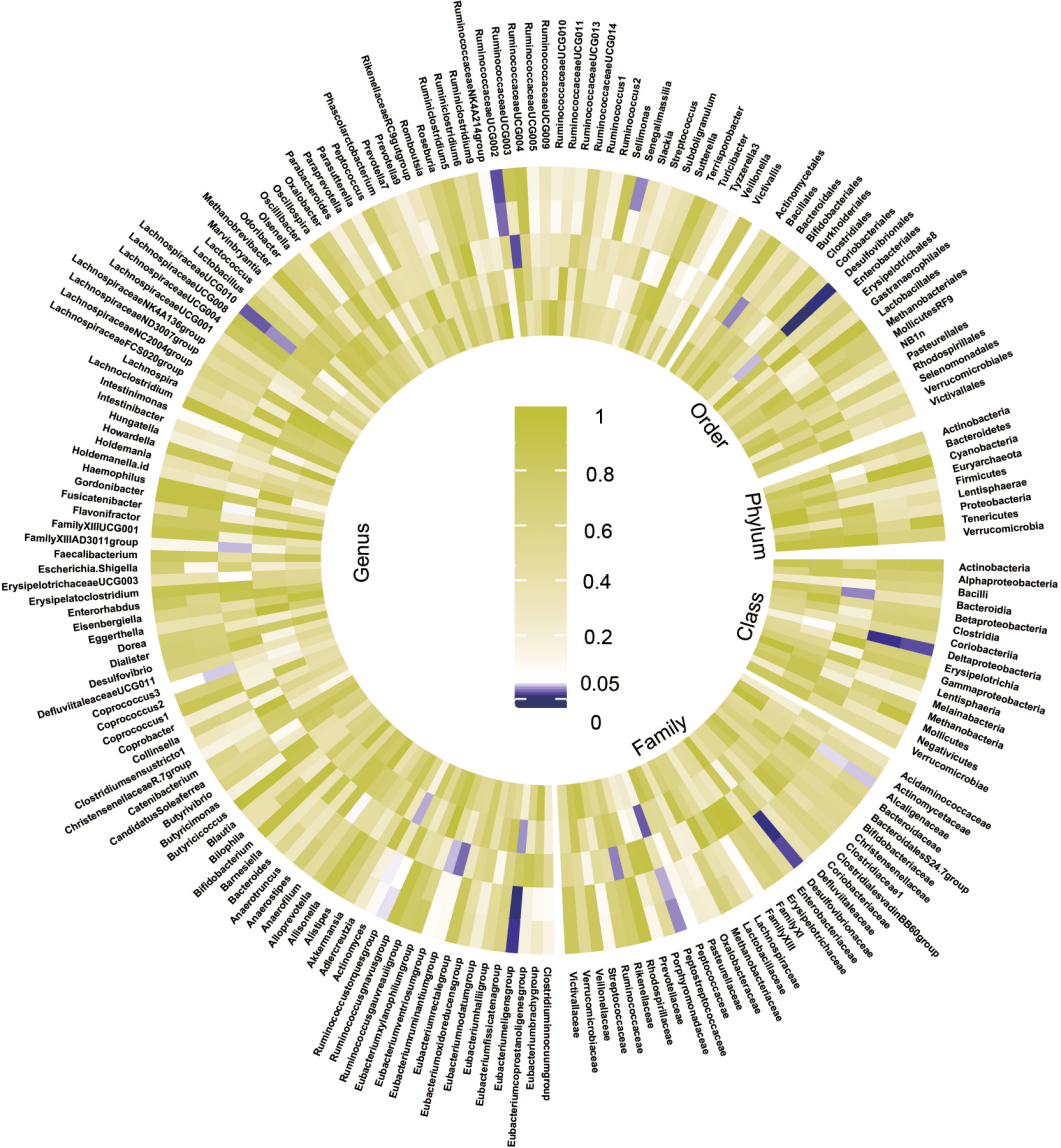
Preliminary MR estimates for the associations between gut microbiota and the risk of CKD. From the inner to outer circles, they represent the estimates of: mode-based estimation, weighted median, MR-Egger, MR-PRESSO and inverse-variance weighted methods, respectively. And the shades of color reflect the magnitude of the p-value.

The preliminary analysis results for the associations between genetically proxied gut bacterial taxa and risk of CKD are provided in **Figure 2** and **Table S5**. All estimates were presented as odds ratios (OR) for per standard deviation (SD) increment in the corresponding exposure.

**Figure 3:**
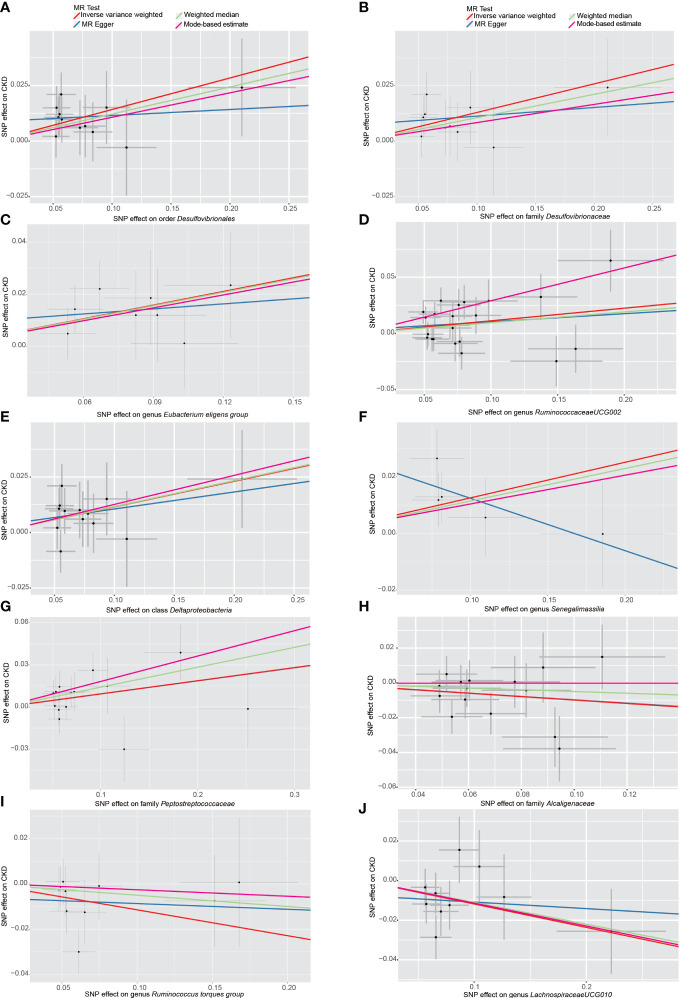
Scatter plots of the MR analyses for the association of 10 gut bacterial taxa and the risk of chronic kidney disease. **(A)** Causal effect of *Desulfovibrionales* on CKD; **(B–J)** Potential causal effect of nine other gut bacterial taxa on CKD. SNP, single nucleotide polymorphisms; MR, mendelian randomization; CKD, chronic kidney disease.

Among the 196 tested taxa phenotypes, after a rigorous Bonferroni correction, a marginal significant causal relationship between order *Desulfovibrionales* and CKD risk was identified by IVW method. To be exact, the genetically predicted higher abundance of order *Desulfovibrionales* in the human gut was causally associated with an increased risk of CKD (IVW OR = 1.15, 95% confidence interval [CI] 1.05-1.26, P = 0.0026). This causal relationship was further confirmed by the MR-PRESSO results, with the significance level reaching the Bonferroni-corrected threshold (OR = 1.15, 95% CI 1.09-1.22, P = 5.5×10^-4^). Causal estimates from WM, MR-Egger and MBE analyses also supported this association in a consistent but non-significant (or nominal significant) direction (**Figure 3A** and **Table 1**). In addition, the statistical power of the causal inference obtained by IVW method was calculated to be 0.93, with a type-I error rate of 0.05.

**Table 1:** Significant and nominal significant MR results.

Exposure	Methods	OR	95% CI	pval	Power
*Desulfovibrionales*	IVW	1.15	1.05-1.26	0.003*	0.93
	WM	1.13	1.01-1.27	0.039	
	MR-Egger	1.03	0.80-1.32	0.836	
	MR-PRESSO	1.15	1.09-1.22	0.001*	
	MBE	1.12	0.93-1.33	0.252	
*Eubacterium eligens group*	IVW	1.19	1.05-1.35	0.006	0.94
	WM	1.19	1.02-1.39	0.031	
	MR-Egger	1.07	0.68-1.68	0.790	
	MR-PRESSO	1.19	1.11-1.28	0.002	
	MBE	1.18	0.94-1.48	0.194	
*Desulfovibrionaceae*	IVW	1.14	1.03-1.26	0.011	0.82
	WM	1.11	0.97-1.27	0.118	
	MR-Egger	1.04	0.81-1.34	0.772	
	MR-PRESSO	1.14	1.07-1.21	0.003	
	MBE	1.09	0.90-1.31	0.408	
*Ruminococcaceae UCG-002*	IVW	1.12	1.03-1.22	0.012	0.96
	WM	1.10	0.98-1.23	0.099	
	MR-Egger	1.08	0.83-1.39	0.592	
	MR-PRESSO	1.12	1.03-2.39	0.020	
	MBE	1.34	1.00-1.79	0.063	
*Deltaproteobacteria*	IVW	1.12	1.03-1.23	0.012	0.82
	WM	1.12	0.99-1.27	0.064	
	MR-Egger	1.08	0.84-1.39	0.570	
	MR-PRESSO	1.12	1.05-1.20	0.005	
	MBE	1.14	0.96-1.36	0.173	
*Lachnospiraceae UCG-010*	IVW	0.89	0.81-0.98	0.016	0.67
	WM	0.90	0.79-1.02	0.090	
	MR-Egger	0.97	0.76-1.23	0.798	
	MR-PRESSO	0.89	0.81-0.97	0.030	
	MBE	0.89	0.74-1.08	0.262	
*Senegalimassilia*	IVW	1.13	1.01-3.39	0.028	0.49
	WM	1.12	0.98-4.39	0.087	
	MR-Egger	0.83	0.59-5.39	0.375	
	MR-PRESSO	1.13	1.01-6.39	0.093	
	MBE	1.11	0.91-1.35	0.369	
*Peptostreptococcaceae*	IVW	1.10	1.01-1.19	0.028	0.7
	WM	1.15	1.03-1.29	0.016	
	MR-Egger	1.10	0.90-1.33	0.366	
	MR-PRESSO	1.10	1.02-1.19	0.036	
	MBE	1.20	0.96-1.49	0.127	
*Alcaligenaceae*	IVW	0.91	0.82-1.00	0.042	0.69
	WM	0.95	0.83-1.09	0.486	
	MR-Egger	0.91	0.61-1.36	0.651	
	MR-PRESSO	0.91	0.83-0.99	0.045	
	MBE	1.00	0.78-1.28	0.988	
*Ruminococcus torques group*	IVW	0.89	0.80-1.00	0.046	0.63
	WM	0.95	0.82-1.11	0.518	
	MR-Egger	0.98	0.72-1.33	0.876	
	MR-PRESSO	0.89	0.81-0.99	0.058	
	MBE	0.97	0.78-1.20	0.800	

*represents that the p-value meets the Bonferroni-corrected significance threshold.

OR, odds ratio; CI, confidence interval; IVW, inverse-variance weighted; WM, weighted median; MR-PRESSO, Mendelian Randomization Pleiotropy Residual Sum and Outlier; MBE, mode-based estimate.

Although the *p* value corresponding to the causal effect estimated by the IVW method (2.6×10^-3^) was still 0.0001 away from the Bonferroni-corrected threshold(2.5×10^-3^), we still considered the conclusion that the order *Desulfovibrionales* has a causal effect on CKD to be reliable. First, the Bonferroni correction is very stringent, and the *p* value of this IVW result was extremely close to the Bonferroni-corrected threshold. Second, the WM, MR-Egger, MR-PRESSO and MBE results were all in the consistent direction as the IVW results, and the *p* value of the MR-PRESSO result even reached the Bonferroni-corrected threshold. Third, the result estimated by IVW method had a high statistical power of 0.93. In fact, as long as we remove even one bacterial order from the analysis of this study, the above *p* value will reach the Bonferroni-corrected threshold, but including as many taxa as possible as exposures allows for a more macroscopic view and judgment of the overall effect of the gut microbiota on CKD.

Additionally, we also detected potential causalities between other 9 taxa and CKD because the IVW analysis results corresponding to these 9 phenotypes were nominal significant (*p* < 0.05, see **Table 1**). As shown in **Figure 3B–J**), among these 9 taxa, *Deltaproteobacteria* (IVW OR = 1.12, 95% CI 1.03-1.23, P = 0.012), *Desulfovibrionaceae* (IVW OR = 1.14, 95% CI 1.03-1.26, P = 0.011), *Peptostreptococcaceae* (IVW OR = 1.10, 95% CI 1.01-1.19, P = 0.028), *Eubacterium eligens group* (IVW OR = 1.19, 95% CI 1.05-1.35, P = 0.006), *Ruminococcaceae UCG-002* (IVW OR = 1.12, 95% CI 1.02-1.22, P = 0.012) and *Senegalimassilia* (IVW OR = 1.13, 95% CI 1.01-1.27, P = 0.028) were identified to have suggestive positive causal effects on the risk of CKD, whereas *Alcaligenaceae* (IVW OR = 0.91, 95% CI 0.82-1.00, P = 0.042), *Lachnospiraceae UCG-010* (IVW OR = 0.89, 95% CI 0.81-0.98, P = 0.016) and *Ruminococcus torques group* (IVW OR = 0.89, 95% CI 0.80-1.00, P = 0.046) had a tendency to causally decrease the risk of CKD. Except for genus *Senegalimassilia*, the results of other MR analyses (WM, MR-Egger, MR-PRESSO and MBE) for the remaining 8 taxa were consistent with their respective IVW results. For *Senegalimassilia*, the effect estimated by MR-Egger was in a reversed direction to the results of the other four MR analyses, although it was not significant. This situation would theoretically require a narrowing of the genome-wide significance threshold followed by a more rigorous selection of IVs for reanalysis (Chen et al., 2021), but this process was not possible because there were few corresponding SNPs left. In the absence of horizontal pleiotropy (see below), the causal effects analyzed by IVW are more precise than those by MR-Egger (Bowden et al., 2016), and therefore the final retention of this IVW estimate for genus *Senegalimassilia* is acceptable.

### 3.3 Sensitivity analyses and detection of potential pleiotropy

In order to avoid excessive bias effects, a series of measures were taken to test the sensitivity of MR analysis and to detect the potential pleiotropy of IVs for each phenotype. A few outliers were found by MR-PRESSO method, and what shown in the **Table S5** are the results after these outliers have been eliminated. It is worth mentioning that no outliers with pleiotropic effect were detected in the IVs of the above 10 taxa that may be causally associated with CKD. Further, as shown in **Table 2**, in these 10 taxa, the Cochran’s Q test indicated that there was no evidence of heterogeneity: all P values were > 0.05. Similarly, the funnel plots were all symmetrical, also indicating no heterogeneity in the results (**Figure S1**). The intercepts of MR-Egger regression showed no sign of horizontal pleiotropy in these 10 taxa (**Table 2**). And the results of the leave-one-out analysis showed that no matter which SNP was removed, it would not have a fundamental impact on the results (**Figure S2**). Equally important, after looking up the PhenoScanner, no SNP associated with confounding factors was found in these 10 taxa (**Table S6**). Last but not least, due to the possible overlap of participants in the exposure and outcome GWAS included in this MR study, we calculated the bias and type I error rates for MR with sample overlap. Since it was not possible to determine the exact number of samples overlapped, we assumed that the maximum overlap exists. The maximum sample size for possible overlap in this study was 2216 (1220 from RS3 and 996 from SHIP, see **Table S2**), with a corresponding overlap proportion of 0.12 (2216/18340). Under this condition, the bias of the MR results was calculated to be less than 0.006 for all phenotypes, with a type I error rate of 0.05. In summary, the results of all these sensitivity analyses reflect the robustness of our MR analyses.

**Table 2:** Sensitivity analysis for significant and nominal significant estimates.

Exposure	Egger intercept	pval	Q	P value	MR-PRESSO global test
*Desulfovibrionales*	0.009	0.355	4.314	0.960	0.964
*Eubacterium eligens group*	0.008	0.638	2.388	0.935	0.940
*Desulfovibrionaceae*	0.007	0.474	3.661	0.932	0.941
*Ruminococcaceae UCG-002*	0.003	0.747	29.425	0.104	0.118
*Deltaproteobacteria*	0.003	0.757	6.230	0.904	0.920
*Lachnospiraceae UCG-010*	-0.008	0.475	7.920	0.542	0.612
*Senegalimassilia*	0.031	0.165	4.839	0.304	0.363
*Peptostreptococcaceae*	0.000	0.996	11.440	0.574	0.565
*Alcaligenaceae*	0.000	0.987	12.014	0.605	0.618
*Ruminococcus torques group*	-0.006	0.560	7.583	0.577	0.568

## 4 Discussion

To date, this is the first MR study to explore whether the gut microbiota has a causal effect on the risk of CKD by using large-scale gut microbiome gene data. In this study, the two-sample MR analysis based on the largest GWAS datasets not only demonstrated that the increased abundance of *Desulfovibrionales* was causally associated with an increased risk of CKD, but also identified another 9 bacterial taxa that may have causal effects on CKD, which reveals the important role played by gut microbiota in the pathogenesis of CKD and is of reference value for further research.

CKD is the fastest-growing cause of kidney-related death in recent years due to its tendency to progress to renal failure in advanced stages (Webster et al., 2017). Severe cardiovascular disease is one of the most serious complications and the most common cause of death in patients with CKD (Gansevoort et al., 2013; Webster et al., 2017). Unfortunately, there is currently no approved treatment to reverse the progression of CKD. When it progresses to end-stage renal failure, renal replacement therapy (peritoneal dialysis, hemodialysis and kidney transplantation) is the only way to sustain patient’s life (Liyanage et al., 2015; Kalantar-Zadeh et al., 2021). Therefore, it is crucial to understand the causes of CKD and carry out primary prevention for the occurrence of CKD.

Current studies indicate that gut microbiota is involved in the pathogenesis of many diseases, such as cancer, metabolic syndrome and mental disorders (Meng et al., 2018; Dabke et al., 2019; Rutsch et al., 2020; Zhao et al., 2021). In addition, gut microbiota-related metabolites, e.g., indoxyl sulfate, p-cresyl sulfate, trimethylamine N-oxide and choline, known as uremic toxins, were found to be associated with the development and progression of CKD (Wu et al., 2011; Sun et al., 2012; Tang et al., 2015; Jia et al., 2019; Krukowski et al., 2022). Therefore, it is reasonable to infer that gut microbiota is related to the pathogenesis of CKD, but this correlation has not been previously proven to be causal.

Here, our study provides an important opportunity to advance the understanding of the causal relationship between gut microbiota and CKD. First, in the absence of evidence from clinical randomized trials and related observational studies, our work used large-scale GWAS data to validate the causal effect of gut microbiota on CKD, providing literature support for further studies in this field. Second, we found that higher abundance of order *Desulfovibrionales* causally predicted a higher risk of CKD. This suggests that it may be feasible to screen patients with CKD by detecting *Desulfovibrionales* in the stool and that high abundance of *Desulfovibrionales* could be regarded as a signal that preventive measures against CKD should be intensified in a timely manner. In addition, our findings suggest that the use of appropriate antibiotics against order *Desulfovibrionales* may be a stratege to prevent CKD. Third, we also found nine additional bacterial taxa that may have causal effects on CKD, whose associations with CKD were nominal significant. Although not reaching Bonferroni-corrected significance, the role of these nine bacterial taxa in the pathogenesis of CKD should not be overlooked. As potential indicators for assessing the risk of CKD, the effects of these taxa on renal function deserve further attention. Interestingly, three of these nine taxa (family *Alcaligenaceae*, genera *Lachnospiraceae UCG-010* and *Ruminococcus torques group*) were found to potentially reduce the risk of CKD, and these results are helpful for clinical translational application. At present, the therapeutic potential of probiotics supplementation for CKD has been demonstrated, and fecal microbiota transplantation has been verified in a mouse model of CKD to reduce the levels of cresol derivatives in the blood and reduce the risk of CKD-related complications (Krukowski et al., 2022). The three taxa identified in this study, which were negatively associated with the risk of cCKD, may be used as oral probiotic supplements for the prevention of CKD in high-risk populations in the future, and may also provide some guidance for further studies related to fecal microbiota transplantation for the treatment of CKD.

Our results are to some extent consistent with the previous studies (Mazidi et al., 2020; Luo et al., 2022). Recently, an MR study observed the effects of two enteropathogenic bacteria (*Shigella* and *Campylobacter*) and *Candida* on CKD, and found that *Candida* was associated with an increased risk of CKD (IVW or = 1.071, P = 0.039) (Luo et al., 2022). Although *Candida* actually belongs to fungi, it can also be regarded as a member of gut microbiota in a broad sense. It is worth mentioning that Mazidi et al. once analyzed the causal effects of 8 bacterial genera on renal function in a MR study, and concluded that genus *Desulfovibrio* had no causal relationship with CKD, but had a causal effect on the estimated GFR (Mazidi et al., 2020). Similarly, the results of our study also pointed out that genus *Desulfovibrio* had no causal effect on CKD. The difference is that our research demonstrated a causal effect of order *Desulfovibrionales* on CKD, and the causal effect of family *Desulfovibrionaceae* on CKD was nominal significant. These results implies that some other bacterial taxa under the taxonomy of *Desulfovibrionales*, rather than *Desulfovibrio*, may have a causal effect on CKD, and that such effect may be an accumulation of multiple taxa’s effects on CKD.

The main weakness of previous MR studies is that only a small number of bacterial taxa were selected as exposures to analyze their relationship with CKD, while we used a way larger scale of gut microbiota data (196 taxa) to more comprehensively analyze the impact of gut microbiota on CKD. what’s more, The GWAS data for CKD used in the two previous studies included a sample size of 133,814 individuals (12,385 cases), whereas the database we used contained approximately 3.3 times the number of cases (n = 41395) and total sample size (N = 480698). Taken together, our study demonstrated the causal effect of gut microbiota, specifically *Desulfovibrionales*, on CKD based on a large sample size, and to some extent validated previous studies. In an RCT, Ebrahim et al. had verified that β -Glucan prebiotics significantly reduced the serum enterogenous uremic toxins levels in patients with CKD (Ebrahim et al., 2022), thus demonstrating that modifying the gut microbiome, promoting specific beneficial taxa as dominant flora may have a good therapeutic effect for the treatment of CKD. Unfortunately, due to the limited study duration and the number of participants, no effects of β -Glucan prebiotics on abundances of genera were observed in this RCT (Ebrahim et al., 2022), and therefore no probiotics with potential efficacy could be identified. Compared with this RCT, our study took a different perspective, using genetic IVs to simulate RCT to identify gut bacterial taxa that could be used as biomarkers for CKD prevention or could be an alternative approach to reduce the risk of CKD.

Although our MR study demonstrated the causal relationship between order *Desulfovibrionales* and CKD, the mechanism involved remained to elucidate. There is a paucity of literature on the pathogenicity of *Desulfovibrionales*. Based on previous studies (Deedwania, 2014; Mazidi et al., 2020), it is hypothesized that production of hydrogen sulfide (H2S), induction of inflammation and induction of elevated cholesterol, may be the mechanisms by which *Desulfovibrionales* mediates CKD pathogenesis. *Desulfovibrionales* metabolizes sulfur-containing substances in the human body and derives H2S, which is a cytotoxic substance and has pro-inflammatory effects (Weglarz et al., 2003; Peck et al., 2019). However, it is still unclear whether gut microbiota-derived H2S causes immune disorders in CKD (Glorieux et al., 2020). In addition, as intestinal colonizing bacteria, the overgrowth of *Desulfovibrionales* undoubtedly results in infections and systemic inflammatory response, which may increase the risk of CKD (Kalantar-Zadeh et al., 2021). There is evidence that *Desulfovibrionales* may promote increased cholesterol absorption (Hu et al., 2022), and elevated cholesterol has been observed to be associated with an increased risk of CKD (Deedwania, 2014). But in general, the mechanism of how *Desulfovibrionales* causes CKD remains unknown and deserves further study.

Currently, MR studies based on gut microbiome GWAS data are increasingly prevalent. Regarding the selection of IVs for each exposure (taxon), two main parameter settings concerning the threshold of LD r^2^ and genetic distance are commonly used: r^2^ < 0.001 with 10,000 kb (Li et al., 2022), or r^2^ < 0.1 with 500 kb (Ni et al., 2021). In this study, we employed the parameter setting of r^2^ < 0.1 with a genetic distance of 500kb to select IVs. To assess the reliability of MR results under such a reduced filtering condition, we additionally tested the MR results using the parameter setting of r2 < 0.001 with 10,000kb genetic distance (**Table S7**). Comparison of the MR results obtained under these two different parameter settings demonstrated consistent and encouraging results from both analyses. Although there is currently no established guideline for setting the filtering conditions for linkage disequilibrium, it can be inferred from the principle of MR that a lower number of IVs may lead to reduced confounders and pleiotropy but poorer statistical power. Conversely, increasing the number of IVs can improve statistical power but also bring more bias and confounding risks. We therefore recommend that researchers carefully consider the trade-off between statistical power and potential bias when selecting the filtering criteria for IVs. It is advisable to explore multiple sets of r^2^ threshold and genetic distance parameters for analysis, which can serve as one of the approaches to verify the robustness of MR results. In addition, caution should be taken when using the meta-GWAS data of gut microbiome publicly released by Kurilshikov A for MR analysis. Specifically, researchers should note that “other allele” is named “reference allele” in this dataset, as it has been observed that “effect allele” and “other allele” were incorrectly switched in some published MR articles related to gut microbiota.

There are also some limitations in this study. Firstly, all GWAS data involved in this study were obtained from the European participants. Therefore, it remains to be verified whether the conclusions of this study can be generalized to other non-European populations. Secondly, since the instrumental variables were derived from a GWAS meta-analysis, we were unable to explore stratification effects and other nonlinear relationships. Although no conclusions have been drawn from observational studies on whether the effect of gut microbiota on the risk of CKD is linear, the possibility of a nonlinear model cannot be excluded. In the future, individual-level GWAS data are expected to be used for nonlinear MR study (Staley and Burgess, 2017) to further explore the nonlinear relationship between gut microbiota and CKD. Despite these potential limitations, we have confirmed by a series of sensitivity analyses that the causal estimates of in this study were robust, i.e., this study accurately reflects the strong association between gut microbiota and CKD risk.

## 5 Conclusion

In conclusion, this study demonstrated the causal effect of gut microbiota (especially order *Desulfovibrionales*) on CKD by MR analysis, which provides theoretical basis for guiding clinical work and may point the way for future research. We hope that doctors and researchers should pay more attention to the monitoring of gut microbiota in the prevention of CKD, so as to discover more risk predictors and potential salutary taxa for renal function, which is the most important clinical significance of this study.

## Data availability statement

The original contributions presented in the study are included in the article/**Supplementary Material**. Further inquiries can be directed to the corresponding authors.

## Ethics statement

No additional ethical approval is required as this is a re-analysis of data that is already publicly available.

## Author contributions

Conceived and designed the experiments: XC, ML, and JC. Performed and analyzed the experiments: XC, ML, JC, and SL. Collecting data: SL and XH. Wrote the manuscript: ML and XC. Read and approved the final manuscript: JC, SL, XH, LX, HL and WF.
