# Supplementary material for: Causal effects of gut microbiota on the risk of chronic kidney disease: a Mendelian randomization study
Source: Front Cell Infect Microbiol. 2023 Mar 31;13:1142140. doi: 10.3389/fcimb.2023.1142140 (PMC10102584; doi:10.3389/fcimb.2023.1142140)
Supplement: Supplementary file 1 [file DataSheet_1.docx]

Supplementary Material

**Causal Effects of Gut microbiota on the Risk of Chronic Kidney Disease: A Mendelian Randomization Study**

**Mingli Luo ^1,2 †^, Jiahao Cai ^3^** **^†^, Shulu Luo ^4^, Xiaosi Hong ^5^, Lingxin Xu ^6^, Honghong Lin ^7^, Xiong Chen ^1 *^, Wen Fu ^1 *^**

*** Correspondence:**

Xiong Chen
[chenx239@mail2.sysu.edu.cn](mailto:chenx239@mail2.sysu.edu.cn)

Wen Fu
lydia_fw@hotmail.com


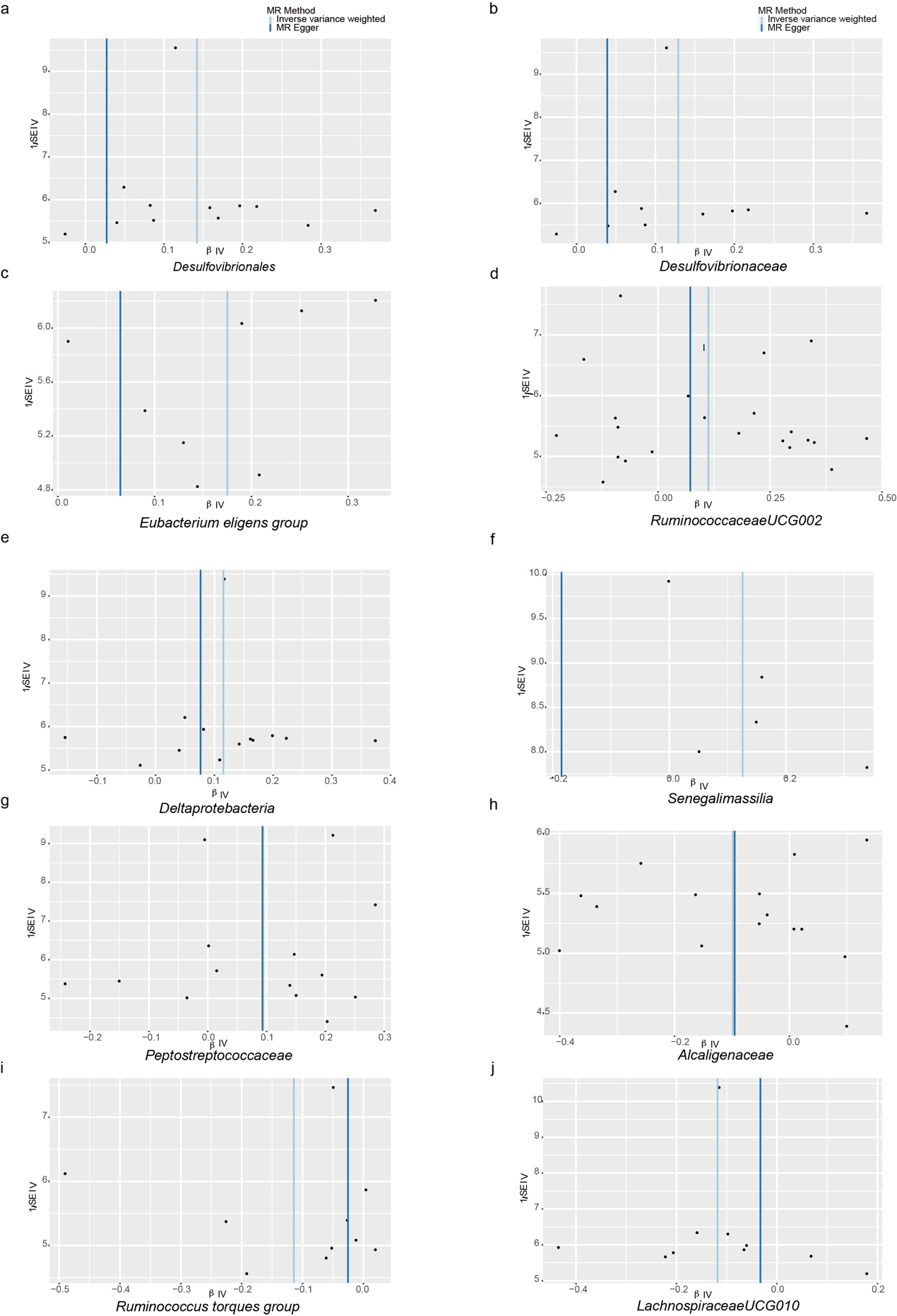


**Figure S1:** Funnel plots from 10 gut bacterial taxa on the risk of CKD.


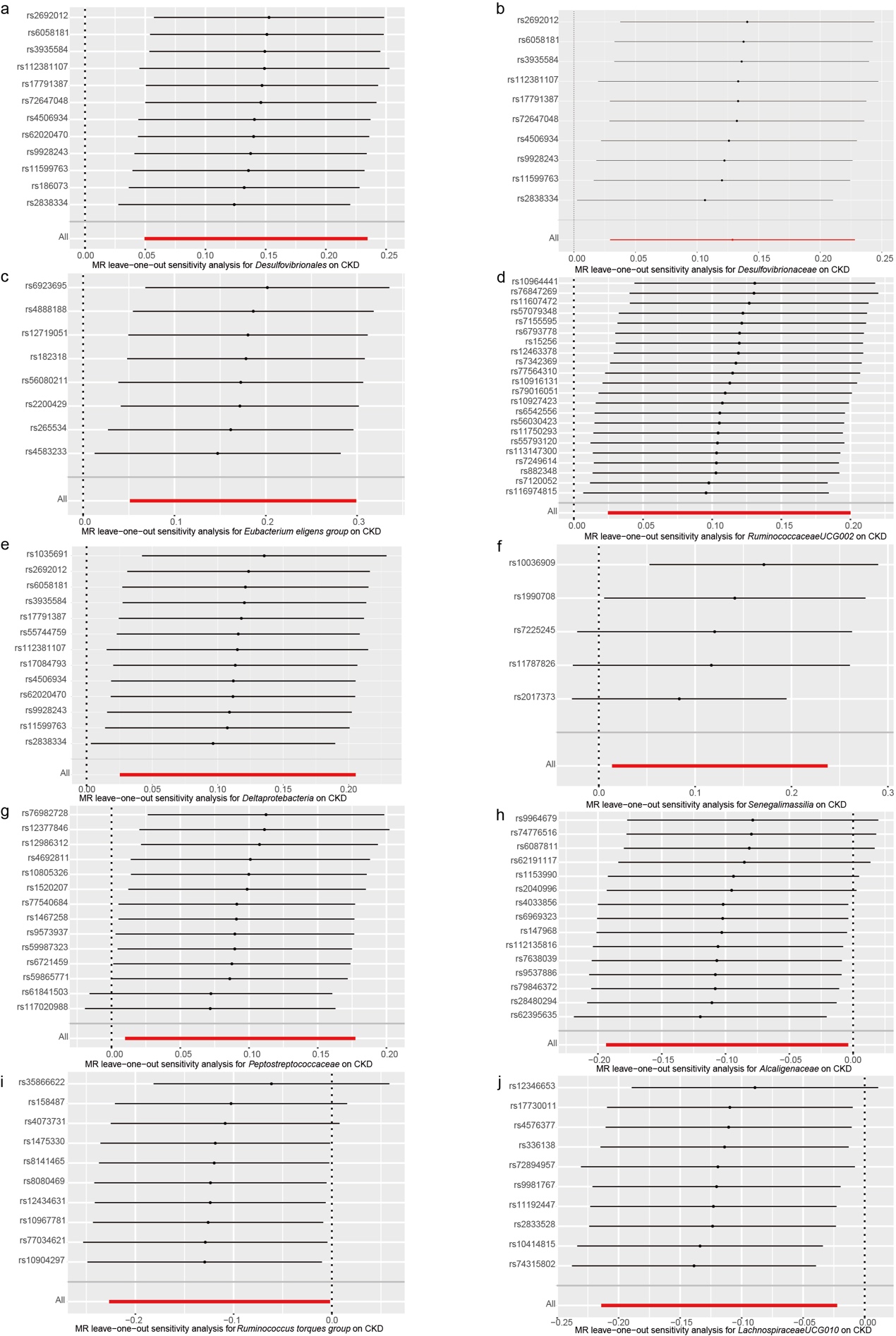


**Figure S2:** Leave-one-out analyses for the causal estimates of 10 gut bacterial taxa on CKD.

MR, mendelian randomization; CKD, chronic kidney disease.
